# Follow-up of collagen crosslink excretion in patients with oral squamous cell carcinoma and analysis of tissue samples

**DOI:** 10.1038/sj.bjc.6601325

**Published:** 2003-10-28

**Authors:** I N G Springer, H Terheyden, M A A Suhr, P Warnke, A Dunsche, M Tiemann, Y Açil

**Affiliations:** 1 Department of Oral and Maxillofacial Surgery, University of Kiel, Arnold-Hellerstr. 16, D-24105 Kiel, Germany; 2 Department of Oral and Maxillofacial Surgery, Klinikum Nord, Hamburg, Tangstedter Landstrasse 400, 22417 Hamburg, Germany; 3 Department of Oral and Maxillofacial Surgery, Städt. Klinikum Karlsruhe, Moltkestr. 90, 76133 Karlsruhe, Germany; 4 Department of Surgical Pathology, University of Kiel, Niemannsweg 11, 24105 Kiel, Germany

**Keywords:** pyridinoline, collagen crosslinks, oral squamous cell carcinoma, tissue samples

## Abstract

The presence of an oral squamous cell carcinoma (OSCC) may be associated with increased urinary excretion of the markers of collagen degradation, hydroxylysylpyridinoline (HP) and lysylpyridinoline (LP). We investigated the possibility of these markers predicting the presence of active disease. Patients from a current study on HP and LP were included as follows: Group 1a (OSCC with confirmed mandibular bony infiltration, *n*=12), group 1b (group 1a patients >6 months after successful treatment), group 2a (OSCC without evidence of mandibular bone infiltration, *n*=8), group 2b (group 2a patients >6 months after successful treatment), group 3a (recurrent OSCC, *n*=8), group 3b (group 3a patients >6 weeks later, symptoms unchanged) and group 4 (control group, *n*=74). Tissue samples from tumour tissue and adjacent healthy mucosa were additionally investigated for HP and LP concentrations (*n*=8). The decrease in the urinary concentrations of HP and LP was statistically significant between groups 1a and 1b (*P*<0.001 for HP and LP), but not between groups 2a and 2b (*P*=0.07 for HP and LP), while values in groups 1b and 2b were within the normal range. When comparing groups 3a and 3b, a significant increase was observed for LP (*P*=0.050), but not HP (*P*=0.208). In conclusion, successful treatment of OSCC with bony involvement may be associated with a reduction of urinary HP and LP, whereas ongoing disease may result in an increase of LP. HP and LP may both be useful markers of tumour progression in patients with OSCC.

Hydroxylysylpyridinoline (HP) and lysylpyridinoline (LP) are two nonreducible crosslinks of mature collagen, which are formed by a sequence of post-translational modifications. HP is a derivative of three residues of hydroxylysine, and is present in virtually all mature tissues (tendon, vessel wall, cartilage, dentine and bone). LP is a derivative of two residues of hydroxylysine and one residue of lysine, and is found primarily in dentine and bone (Body and Delmas, 1992; Eyre, 1992; Miyamoto *et al*, 1994; Acil *et al*, 1996, 2002a, 2002b; Papatheofanis, 1997; Tamada *et al*, 2001; Jepsen *et al*, 2003; Springer *et al*, 2003a, 2003b).

It has been suggested that the detection of LP in the serum or urine may be a helpful marker in establishing and possibly quantifying bone matrix resorption (Body and Delmas, 1992; Miyamoto *et al*, 1994; Vinholes *et al*, 1996; Papatheofanis, 1997; Tamada *et al*, 2001; Springer *et al*, 2003a). In a previous study, we were able to assess the range and upper limit (HP_max_ and LP_max_) of normal values. We were able to show that the measurement of LP in the urine was able to separate a group of patients with oral squamous cell carcinoma (OSCC) with bone infiltration from patients with OSCC without bone infiltration with a sensitivity of 100% and a specificity of 100% (Springer *et al*, 2003a). In that paper, we suggested that when the level of LP exceeds LP_max_ in a patient with a confirmed OSCC, bony invasion by the malignant process is highly likely and further investigations to confirm this should be performed. We also suggested that a urinary level of LP less than LP_max_ in patients with OSCC obviated the need for such investigations (Springer *et al*, 2003a). We found that urinary HP is not specific for bony invasion, but may nonetheless be increased in the urine of patients with bony metastases as compared to patients without bony metastases. The average urinary HP concentration is also higher in patients with OSCC without mandibular bony invasion than in controls (Springer *et al*, 2003a). We were able to demonstrate that increasing or increased values of HP and LP are associated with the presence of tumour tissue. The presence of such tissue could be detected with a sensitivity of 90% and specificity of 65% whether bone was infiltrated or not (Springer *et al*, 2003a). The urinary levels of HP and, in particular, LP have been shown to be increased in patients with bony metastases from multiple myeloma, carcinoma of the breast, lung, prostate gland, kidney, throat and digestive tract (Takeuchi *et al*, 1996; Papatheofanis, 1997; Walne *et al*, 1997; Yoshida *et al*, 1997; Coleman *et al*, 1999; Marttunen *et al*, 1999; Tamura *et al*, 1999; Woitge *et al*, 1999, 2001; Demers *et al*, 2000; Fontana and Delmas, 2000; Liubimova *et al*, 2000; Izumi *et al*, 2001; Tamada *et al*, 2001).

Conventional tumour markers are released by neoplastic tissue, progressively dedifferentiated subclones may not necessarily express the same specific markers (Mendelsohn, 1995). The aim of this study was to evaluate the predictive value of a reduction in HP and LP in patients with OSCC after successful treatment and of constant or increasing values in patients with ongoing disease.

We were also interested in further investigating the origin of increased urinary concentrations of HP and LP in patients with OSCC. For this purpose, tissue samples were analysed. While tumour markers, in general, are specific for certain tumours and are presumed to be released from neoplastic tissue (Mendelsohn, 1995), HP and LP indicate the destruction/resorption of healthy mature collagen in the surrounding tissue. To the best of our knowledge, no analysis of the concentrations of HP and LP in carcinomatous tissue for intraindividual comparison with normal tissue has been performed to date.

Patients included in our previous study were followed up in order for us to observe intraindividual variations in urinary HP and LP excretion. The authors hypothesised that a successful treatment of patients with OSCC with and without bony infiltration leads to a reduction of urinary HP and LP concentrations toward the normal range, and that ongoing disease might be associated with constant or increasing values. Furthermore, the authors hypothesised that increased values of urinary HP and LP in patients with OSCC are due to the resorption of mature collagen in tissues adjacent to the carcinoma.

## MATERIALS AND METHODS

Patients were recruited from the Cancer Clinic in the Department of Oral and Maxillofacial Surgery, University of Kiel, Germany. We obtained 56 urinary samples from 28 patients (age range 44–88 years). The controls were those used in our previous paper (Springer *et al*, 2003a).

*Group 1a (n*=*12)*: Samples of patients with OSCC (pT4 N1-2 M0) with bone infiltration (eight females, four males; six patients 49–60 years of age; six patients 61–88 years of age).
*Group 1b (n*=*12)*: Samples of patients of group 1a at least 6 months after successful treatment with no sign of recurrence.
*Group 2a (n*=*8)*: Samples of patients with OSCC (pT1-3 N0-1) with no bony infiltration (one female, seven males, two patients 44–60 years of age; six patients 61–81 years of age).
*Group 2b (n*=*8)*: Samples of patients of group 2a at least 6 months after successful treatment with no sign of recurrence.
*Group 3a (n*=*8)*: Samples of patients with disease recurrence where no treatment was undertaken (four females, four males; five patients 52–60 years of age; three patients 61–83 years of age).
*Group 3b (n*=*8)*: Samples of patients of group 3a more than 6 weeks later, symptoms unchanged, where no treatment had been performed.
*Group 4 (n*=*74)*: Control patients without disease (53 males, 21 females; 28 patients 36–60 years of age; 56 patients 61–91 years of age) as described elsewhere (Springer *et al*, 2003a).

As stated in our previous paper, patients who were seen in our regular follow-up programme after apparently adequate initial treatment of OSCC were included as controls, if treatment had been completed more than a year prior to entry into the study. In this group, an *R*_0_-resection and<T2-disease were required for inclusion as a control. Patients who had had a history of malignancy other than OSCC or had documented alterations in renal function (urea >50 mg dl^−1^, creatinine >1.2 mg dl^−1^) were excluded. Recall patients were excluded if a prior recurrence had been documented and successfully treated. Patients were also excluded if they had had a surgical procedure or a trauma less than 6 months prior to entry into the study (see Results). Patients in groups 1a, 2a and 3a were staged by ultrasound and computed tomography. Technetium 99m methylene diphosphonate (MDP) bone scans with planar imaging and single-positron emission computed tomographies (SPECTs) were additionally performed on all patients included in the study (with the exception of the controls). A chest X-ray, abdominal ultrasound, endoscopic examination of the upper aerodigestive tract and gynaecological investigations in female patients completed the invesitagions for a concomitant disease process. The histopathological examination was performed by a single pathologist. Patients of groups 1b, 2b and 3b were staged by ultrasound and clinical examination only.

### Tissue samples

Tissue samples were taken from tumour tissue and adjacent healthy mucosa for intraindividual comparison (*n*=8). Resection margins were left undisturbed for histographic control.

### Preparation and hydrolysis of urine

Samples were taken in the morning and stored at −70°C until further processing. All laboratory investigations were performed in a single laboratory. HP and LP levels seem to be stable in urine samples for over 10 years if samples are stored at this temperature. The urine samples were centrifuged at 1000 r.p.m. for 5 min. A measure of 2 ml of supernatant was lyophilised and subsequently redissolved in 2 ml 6 N hydrochloric acid. The samples were hydrolysed at 110°C for 24 h and centrifuged at 1000 r.p.m. for 5 min. A volume of 1 ml of each hydrolysate was added to a mixture of 1 ml glacial acetic acid, 2 ml *n*-butan-1-ol and 5 ml 10% CF-1-slurry (fibrous cellulose powder, Whatman, Maidstone, England). The CF-1-slurry was composed of 10% (w v^−1^) CF-1 in a mobile phase containing *n*-butan-1-ol, glacial acetic acid and water (4 : 1 : 1). A column was prepared by adding the mixture of hydrolyzate and CF-1-slurry as described above to an econo-column polyprop (40 × 8 mm^2^, Bio-Rad München, Germany). The resin was washed three times with 5 ml of the mobile phase. Subsequently, the pyridinium-containing eluate was eluted from the column with 3 × 2 ml distilled water into a 15 ml plastic tube and traces of *n*-butan-1-ol were removed from the surface of the eluate. Thereafter, the lyophilised eluate was redissolved in 1 ml 0.22% (v v^−1^) *n*-heptafluorobutyric acid (HFBA) and centrifuged at 1000 r.p.m. for 5 min. A volume of 200 *μ*l of the sample were analysed by HPLC (as below). The variations within and between series were 2 and 4.8%, respectively.

### Preparation and hydrolysis of biopsies

Preparation and analysis of tissue samples was performed as described in our previous studies (Acil *et al*, 2002b; Jepsen *et al*, 2003). Tissue samples were subsequently dissolved in 1 ml 6 M hydrochloric acid and hydrolysed at 110°C for 24 h and centrifuged at 1000 r.p.m. for 5 min. A volume of 1 ml of each hydrolysate was added to a mixture of 1 ml acetic acid, 2 ml *n*-butan-1-ol and 5 ml 10% CF-1-slurry (fibrous cellulose powder, Whatman, Maidstone, England). Further preparation was performed as described for the urine samples above.

### Pyridinoline standards

The HP and LP were quantified by external standards gained from a commercially available adult bovine bone gelatin (Deutsche Gelatine-Fabriken Stoess, Eberbach/Baden, Germany) prior to the application of the samples to the chromatography system. HP and LP were purified by a preparative reverse-phase-column HPLC and the degree of purity was verified by amino-acid analysis (>98% of dry weight) according to a method previously described. (Acil and Müller, 1994; Acil *et al*, 1996, 2000) Serial dilutions of HP between 0 and 2250 pmol nmol^−1^ and LP 0 and 1200 pmol ml^−1^ were analysed to demonstrate the linear response of the external standards.

### Analysis of HP and LP by reverse-phase-column HPLC

Chromatography was performed on a Dionex HPLC system (Idstein, Germany) at 22°C. The flow rate was 0.7 ml min^−1^ using two continually degassed solvents: (A) 0.22% (v v^−1^) HFBA in water and (B) 0.22% (v v^−1^) HFBA in 80% (v v^−1^) acetonitrile. The resin (Inertsil ODS-3 5 *μ*m, 125 × 4.6 mm^2^ C_18_) was equilibrated with 18/82% solvent B to solvent A prior to the application of the sample (200 *μ*l in 0.22% (v v^−1^) HFBA in water). The column was washed with 18/82% (v v^−1^; B/A) for 5 min and developed with the following step gradients:

1. Solvent B (18–20%) over 20 min; the peaks of HP and LP were eluted at approximately 18 and 20 min.
2. Solvent B (20–25%) in 4 min.
3. Solvent B (25–100%) in 1 min plus washing of the column for another 5 min with 100% solvent B.
4. In all, 100–18% over solvent B in 4 min, and 1 min was used for column equilibration, thereafter. The next sample was injected after 35 min.

Fluorescence measurements were obtained with an excitation wavelength of 297 nm and emission wavelength of 397 nm and the concentrations of HP and LP expressed in pmol ml^−1^. After dilution (1 : 20) of 1 ml of patient urine, the urinary creatinine content was measured by the colorimetric Jaffé-reaction and expressed in mg dl^−1^ (Beckman Creatinine Analyzer 2, USA). The urinary content of HP and LP was expressed in relation to the urinary creatinine concentration, that is, in nmol mmol^−1^ creatinine.

### Statistical analysis

There was no statistically significant difference between the sexes, and a normal distribution of the urinary concentrations of HP and LP was seen in the different experimental groups. The arithmetic mean of the urinary levels of HP and LP of the control group (group 4) was calculated and 1.96 s.d. used to define the normal range. These upper limits were called HP_max_ and LP_max_ (Springer *et al*, 2003a). A Wilcoxon matched pair signed-rank test was performed to evaluate the statistical significance of differences between groups 1a and b, 2a and b as well as 3a and b. To evaluate the statistical significance of the difference of the concentration of HP and LP in tissue samples taken from healthy mucosa and carcinoma tissue, the Wilcoxon matched pair signed-rank test was performed. Finally, the average urinary concentrations of HP and LP of groups 1a, 2a, 3a, 1b, 2b and 3b were compared with the control group and a two-tailed Mann–Whitney *U*-test was used to determine the significance of differences between these groups (level of significance *P*<0.05).

### Ethics

The study was conducted in accordance with the standards of the Ethics Committee of the University of Kiel (chairman: Jürgen Schaub, MD, PhD, Professor for Pediatrics, Head of the Department of Pediatrics, University of Kiel, Germany; registration number of the present study: AZ D 309/01) and with the Helsinki Declaration of 1983. The patients were informed about the aim and design of the study and written consent was obtained.

## RESULTS

**Figure 1:**
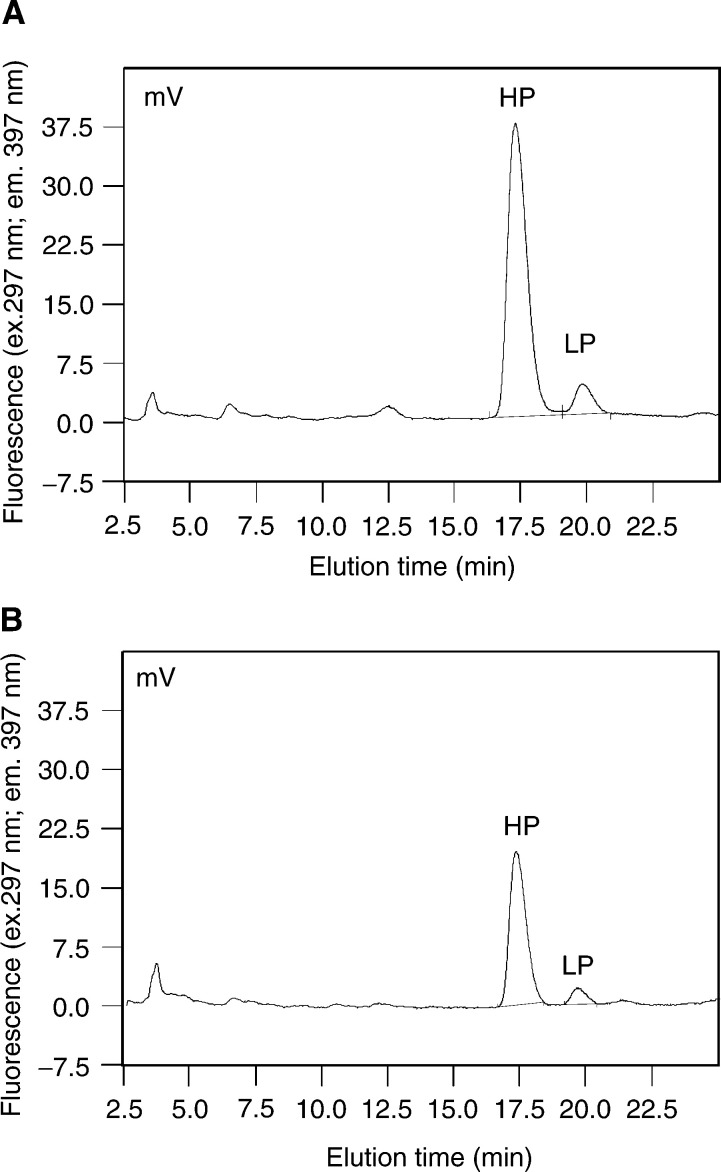
Chromatograms of a patient of group 1a (OSCC with mandibular bony infiltration) at the time of diagnosis (**A**) and group 1b (6 months after successful treatment) (**B**). The fluorescence was monitored with excitation at 297 nm and emission at 397 nm. The HP peak arose at 17.5 min after injection, followed by the LP peak. Of note are the decreased HP and LP peaks in group 1b as compared to group 1a.

The decrease in urinary HP and LP concentrations was statistically significant between groups 1a and 1b (*P*<0.001 for HP and LP, Figure 1

**Table 1:** Urinary concentrations (nmol mmol^−1^ creatinine)

		***n***	**Mean**	**s.d.**	**Min**	**Max**	**25th p**	**Median**	**75th p**
HP	Group 1a	12	**136.70**	52.86	56.00	212.50	90.18	**145.50**	181.05
	Group 1b	12	**64.62**	34.08	13.80	128.20	39.98	**58.90**	85.25
	Group 2a	8	**76.81**	41.58	23.10	141.30	37.75	**75.05**	106.50
	Group 2b	8	**37.59**	18.65	16.60	70.83	21.03	**32.05**	51.38
	Group 3a	8	**117.02**	58.35	49.21	222.50	69.63	**97.06**	161.62
	Group 3b	8	**134.26**	47.66	71.06	193.94	98.82	**118.94**	186.30
	Group 4	74	**50.34**	20.47	13.20	94.10	36.02	**48.85**	66.00
									
LP	Group 1a	12	**30.86**	7.30	21.02	40.43	23.50	**31.92**	38.02
	Group 1b	12	**12.84**	6.49	4.72	22.80	8.00	**9.82**	18.90
	Group 2a	8	**11.93**	5.61	5.50	19.73	5.90	**12.67**	17.56
	Group 2b	8	**8.53**	3.85	2.97	13.83	5.48	**8.02**	12.22
	Group 3a	8	**23.95**	5.60	14.37	34.13	21.30	**23.84**	26.41
	Group 3b	8	**31.42**	7.65	23.27	48.43	26.91	**29.36**	33.43
	Group 4	74	**9.32**	4.43	1.80	19.10	5.67	**8.85**	12.58

**Figure 2:**
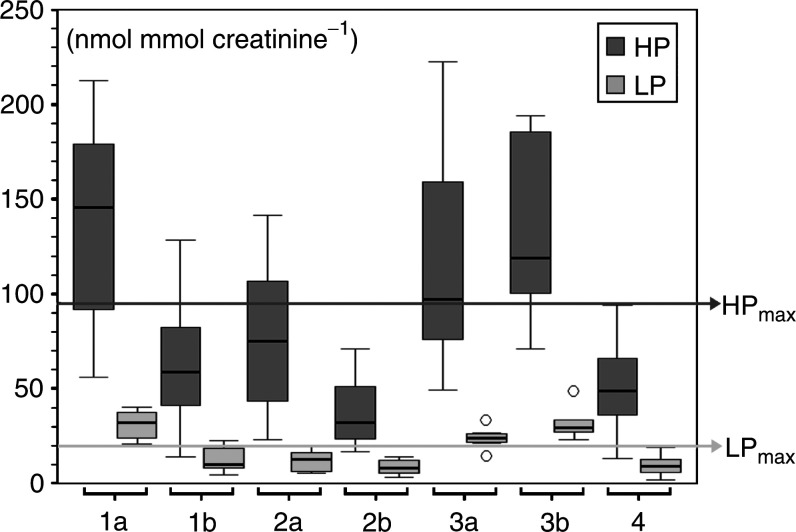
Boxplot, urinary concentrations of HP and LP: each box shows the median, quartiles and extreme values. Group 1a (OSCC with mandibular bony infiltration, *n*=12), group 1b (samples of patients of group 1a >6 months after successful treatment), group 2a (OSCC without mandibular bone infiltration, *n*=8), group 2b (samples of patients of group 2a >6 months after successful treatment), group 3a (recurrence of OSCC, *n*=8), group 3b (samples of patients of group 3a >6 weeks later, symptoms unchanged) and group 4 (control group, patients without disease *n*=74). Lines mark HP_max_ (95 nmol mmol^−1^ creatinine) and LP_max_ (20 nmol mmol^−1^ creatinine). In regard to groups 1a and 2a, the LP_max_ line separates the LP values completely. (○) Values of patients that significantly exceeded the normal range in the recurrence group. While values in groups 1b and 2b approach the normal range as given by group 4 values exceeded it in groups 3a and 3b.

Group 1a=OSCC with mandibular bony infiltration; group 1b=patients in group 1a >6 months after successful treatment; group 2a=OSCC without mandibular bony infiltration; group 2b=patients in group 2a >6 month after successful treatment; group 3a=untreatable recurrence of OSCC; group 3b=patients in group 3a at least 6 weeks later, symptoms unchanged and group 4=control group, patients without disease *n*=74. s.d.=standard deviation, min=minimum, max=maximum, p=percentile.

), but not between groups 2a and 2b (*P*=0.07 for HP and LP). As compared to group 4 (controls), the urinary concentrations of all patients in group 2b were within the normal range while two values for both LP and HP in group 1b were higher than HP_max_ and LP_max_ (Figure 2

). Urinary concentrations of HP of groups 1a and 1b overlapped in five out of 12 patients, while urinary concentrations of bone-specific LP overlapped in only two out of 12 patients. When comparing the urinary concentrations of group 3a with group 3b, a further significant increase was observed for LP (*P*=0.050), but not HP (*P*=0.208) with *both* HP *and* LP being increased further in two out of eight patients. No statistically significant difference was found between groups 1b (follow-up of patients with OSCC with bony infiltration after apparently successful treatment) and 4 (controls, *P*=0.195 for HP and *P*=0.107 for LP) as well as groups 2b (follow-up of patients with treated for OSCC without bony infiltration) and 4 (control,*P*=0.077 for HP and *P*=0.68 for LP). The differences between groups 3a (recurrence group) and 4 (controls, *P*<0.001 for HP and *P*<0.001 for LP) as well as 3b (follow-up of patients with recurrence) and 4 (control, *P*<0.001 for HP and *P*<0.001 for LP) were statistically significant. The average urinary concentration of HP (*P*<0.05) but not LP (*P*=0.20) was significantly increased in group 2a as compared to group 4. The average urinary levels of HP and LP in nmol mmol^−1^ creatinine are shown in Figure 2. Details are provided in Table 1 .

**Figure 3:**
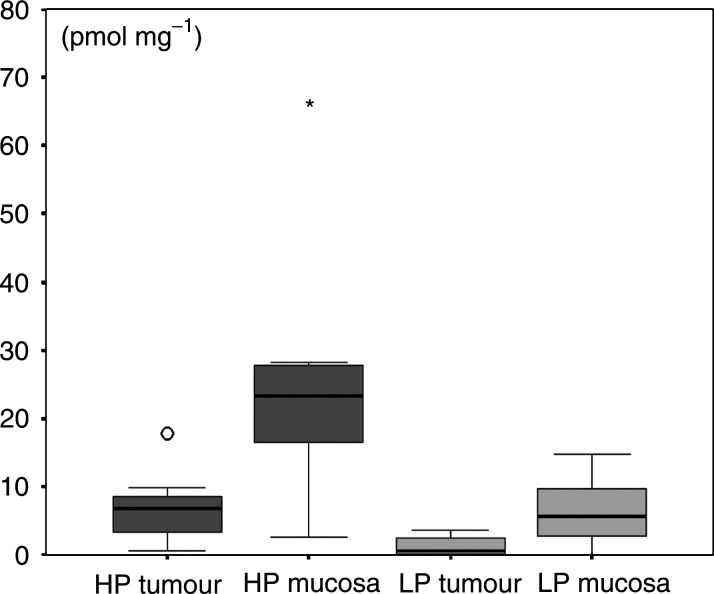
Boxplot of concentrations of HP and LP in carcinoma tissue (*n*=8) and normal mucosa (*n*=8) (pmol g^−1^): A sample of normal appearing mucosa as well as a sample of tumour tissue was obtained in eight patients. Each box shows the median, quartiles and extreme values. The concentrations of both HP (*P*=0.027) and LP (*P*=0.017) were significantly decreased in carcinomatous tissue. It is remarkable that there is a minor concentration of LP in some of the samples. ○ and ^*^ indicate extreme values.

The concentrations of both HP (*P*=0.027) and LP (*P*=0.017) were significantly lower in carcinomatous tissue as compared to healthy mucosa. In six out of eight tissue samples and in seven out of eight mucosa samples, minor concentrations of LP were found (Figure 3

, Table 2

**Table 2:** Concentrations of HP and LP in carcinomatous tissue (*n*=8) and normal mucosa (*n*=8) (pmol g^−1^)

		***n***	**Mean**	**s.d.**	**Min**	**Max**	**25th p**	**Median**	**75th p**
HP	Normal mucosa	8	**25.46**	18.51	2.57	66.11	14.50	**23.23**	27.96
	Carcinoma	8	**6.98**	5.27	0.62	17.82	2.85	**6.87**	9.12
									
LP	Normal mucosa	8	**6.34**	5.00	0.00	14.79	2.13	**5.58**	10.58
	Carcinoma	8	**1.26**	1.48	0.00	3.62	0.08	**0.63**	2.95

A sample of normal appearing mucosa and a sample of tumour tissue was obtained in eight patients. The concentrations of both HP (*P*=0.027) and LP (*P*=0.017) were significantly reduced in carcinoma tissue. It is remarkable that there is a minor concentration of LP in some of the samples.

).

## DISCUSSION

We set out to assess the ability of a decrease of urinary HP and LP concentrations in patients with OSCC after successful treatment, and the value of constant or increasing values in patients with ongoing disease. We were able to show that the decrease in urinary HP and LP concentrations was statistically significant between patients with OSCC with bony infiltration and 6 months after successful treatment for this disease (*P*<0.001 for HP and LP). The authors of the present study would like to point out that urinary concentrations of HP of groups 1a and 1b overlapped in five out of 12 patients, while urinary concentrations of bone-specific LP overlapped in only two out of 12 patients. The reduction of urinary concentrations of LP after successful treatment appears to be of greater significance when compared to the reduction of urinary concentrations of HP. However, a Wilcoxon matched pair signed-rank test was employed to test for significant differences of urinary concentrations of the patients in the course of an *intraindividual* follow-up. The significance of intraindividual differences of a group of patients is addressed by this test, implying that an overlap of the range of urinary concentrations of HP and LP between groups 1a and 1b is not a primary issue. The Wilcoxon matched pair signed-rank test showed intraindividual differences of urinary concentrations of HP and LP to be highly significant (*P*<0.001 for HP and LP).

The differences between patients with OSCC without bony infiltration and values of these patients 6 months after successful treatment were present, but not statistically significant (*P*=0.07 for HP and LP). After successful treatment, values of all patients with OSCC without bony infiltration were within the normal range. Two values for both LP and HP of patients with OSCC with bone infiltration 6 months after successful treatment were greater than HP_max_ and LP_max_, but were significantly decreased as compared to the values at the time of diagnosis. Further increases of LP values and constant HP values in patients with ongoing disease indicated the sensitivity of the assay. As we were able to show in our previous study, increased or increasing values of urinary HP and LP are closely associated with the presence of tumour tissue. The presence of such tissue could be detected with a sensitivity of 90% and specificity of 65% (Springer *et al*, 2003a). The results of this study suggested that urinary HP and LP concentrations normalise in patients after successful treatment and increase in patients with ongoing disease.

Many markers have been evaluated for OSCC, some of which were tissue polypeptide antigen (TPA), carcinoembryonic antigen (CEA), surface antigen, 100 Da (S-100), carbohydrate antigen 19-9 (CA 19-9), CA 125, CA 15-3, squamous cell carcinoma antigen (SCCA), immunosuppressive acidic protein (IAP), alpha-foetoprotein (AFP) and ferritin (FER) (Zoller *et al*, 1990; Kurokawa *et al*, 1993; Kuo *et al*, 1999; Hofele *et al*, 2002). TPA, CEA, CA 19-9 and CA125 levels were analysed in a group of patients with laryngeal or oral cancer pre- and post-therapy (Kuo *et al*, 1999). Only TPA and CEA levels decreased significantly after therapy but clinical use in the disease was described to be limited (Kuo *et al*, 1999). Another study analysed serum levels of CEA, SCCA, IAP, AFP, FER and CA 19-9 in patients with primary OSCC (Kurokawa *et al*, 1993). The positive rates were reported to be 34.5% for CEA, 41.4% for SCCA, 51.7% for IAP, 0% for AFP, 10.3% for FER and 6.9% for CA 19-9, and it was concluded that only a combination of the analysis of CEA, SCCA and IAP could be of some value in the diagnosis of OSCC (Kurokawa *et al*, 1993). Another study did show that the diagnostic value of the tumour markers CEA, Ca 19-9, Ca 125, Ca15-3 exhibited a poor sensitivity in the follow-up of squamous cell carcinoma of the head and neck (Zoller *et al*, 1990).

The detection of epithelial tumour RNA in plasma from colon cancer patients is associated with advanced stages and circulating tumour cells (Silva *et al*, 2002). Two different studies regarding patients with head and neck squamous cell carcinoma (HNSCC) and OSCC, respectively, found that serum p53 antibody is a significant prognostic factor for nodal metastasis (Chow *et al*, 2001; Hofele *et al*, 2002).

CYFRA 21-1 but not CYFRA 8/18 serum levels were suggested to be significantly higher in patients with squamous cell carcinoma of the head and neck as compared to a control group and cutoff values were determined (Niemann *et al*, 1997; Maass *et al*, 1999).

Serum vascular endothelial growth factor (s-VEGF) levels were shown to be significantly increased in patients with advanced laryngeal carcinoma as compared to healthy controls (Teknos *et al*, 2002). There were certain indications that elevated pretreatment s-VEGF levels might indicate a more aggressive disease state and a poorer overall survival in laryngeal carcinoma (Teknos *et al*, 2002). Also, it could be shown that OSCC is associated with significantly increased s-VEGF concentrations and it was suggested that the measurement of the s-VEGF concentration may be helpful to distinguish OSCC patients from healthy individuals (Shang *et al*, 2002).

In contrast to HP and LP, conventional markers such as those named above are in general specific for a certain tumour disease and are thought to be released by neoplastic tissue (Mendelsohn, 1995). Dedifferentiated subclones of the tumour may not necessarily express the same specific marker (Mendelsohn, 1995). Carcinomas are epithelial in origin (Reichart and Philipsen, 1999) and therefore the direct release of HP and LP, which are crosslink residues of mature collagen, is highly unlikely.

To the best of our knowledge, the analysis of the concentration of HP and LP in carcinomatous tissue for intraindividual comparison with normal tissue has not been performed in any kind of neoplasm. We were able to show that the concentrations of HP and LP is significantly decreased in carcinoma tissue as compared to mucosa (*P*=0.027 for HP and *P*=0.017 for LP). We suggest that the destruction of healthy mature collagen in the course of tissue invasion by OSCC tissue is responsible for the release of increased amounts of HP and LP and that therefore urinary HP and LP are progression/invasion markers of OSCC. An advantage in using HP and LP in the follow-up of patients with OSCC is that the sensitivity of these markers is independent of the state of differentiation of the carcinoma.

Minor concentrations of LP in both tumour tissue and healthy tissue indicate that it is not completely specific for dentin and bone as indicated earlier (Body and Delmas, 1992; Miyamoto *et al*, 1994; Vinholes *et al*, 1996; Papatheofanis, 1997; Tamada *et al*, 2001; Acil *et al*, 2002b; Springer *et al*, 2003a, 2003b). The measurement of LP in the urine has been shown to be 100% sensitive and 100% specific in indicating whether bone is invaded by an OSCC or not (Springer *et al*, 2003a). We suggest that low concentrations of LP in healthy mucosa and carcinoma tissue have no impact on the clinical application of the assay in staging and the follow-up of OSCC.

The cost of detection of urinary HP and LP is low when performed in a clinical laboratory on a routine basis (Springer *et al*, 2003a). We suggest that HP and LP could serve as markers of tumour progression, as urinary levels return to normal after successful treatment and increase or remain elevated in patients with a confirmed tumour recurrence. The analysis of the total urinary HP and LP by the HPLC method as applied in the present study could be helpful for intraindividual follow-up of patients with OSCC. We suggest that an intraindividual increase of HP and LP values even within the normal range should at least alert the clinician to the possibility of a recurrence. As we have shown in our previous study, urinary HP and LP concentrations are nonspecific in patients with a previous diagnosis of OSCC. After a patient has been treated for an OSCC, recurrence can be detected with a sensitivity of 90%. While false negatives were not observed, false positives (ca. 18%) have no harmful consequence (Springer *et al*, 2003a). In addition to the clinical examination and ultrasonography, the urinary assay of HP and LP may be valuable in indicating the presence of recurrence in the course of intraindividual follow-up.
